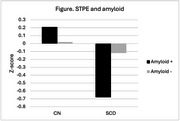# Practice effects in Subjective Cognitive Decline and Biomarker Positivity

**DOI:** 10.1002/alz70857_103148

**Published:** 2025-12-25

**Authors:** Kevin Duff, Jace B King, John M. Hoffman, Dustin B. Hammers

**Affiliations:** ^1^ Oregon Health & Science University, Portland, OR, USA; ^2^ NIA‐Layton Aging & Alzheimer's Disease Research Center, Portland, OR, USA; ^3^ University of Utah, Salt Lake City, UT, USA; ^4^ Huntsman Cancer Institute, University of Utah, Salt Lake City, UT, USA; ^5^ Indiana University School of Medicine, Indianapolis, IN, USA

## Abstract

**Background:**

Subjective cognitive decline (SCD), defined as cognitive complaints in the absence of objective cognitive impairments, may reflect the earliest manifestation of preclinical Alzheimer's disease (AD). However, individuals with SCD are a heterogenous group with various etiologies. Building on our prior work examining short‐term practice effects (STPE) in Mild Cognitive Impairment (MCI) and AD, we examined if STPE in SCD were associated with biomarker positivity.

**Method:**

75 participants classified as cognitively unimpaired according to the Jak/Bondi criteria were divided by whether they presented with cognitive complaints (i.e., SCD, *n* = 11) or not (i.e., CN, *n* = 64). They completed a brief cognitive battery twice across one week to quantify STPE. They also completed a blood draw for APOE genotyping, an MRI for hippocampal volumes, and a PET for amyloid deposition.

**Result:**

For APOE, there was no relationship between STPE and *ε*4 status in those without cognitive complaints (*r* = .06), but the greater number of *ε*4 alleles was associated with smaller STPE in those with SCD (*r* = ‐.42). For MRI, larger hippocampal volumes were associated with larger STPE (*r* = .25). For amyloid PET, 19% of those without cognitive complaints were amyloid positive, whereas 73% of those with SCD were amyloid positive, which is statistically significantly different (*p* < 0.001, see Figure).

**Conclusion:**

Similar to our prior work in MCI and AD, STPE were associated with three biomarkers of AD in the expected direction (i.e., smaller STPE associated with worse biomarker outcomes), especially in those with SCD. Although these findings need to be replicated in a rigorous, prospective study with a larger and more diverse sample, STPE seems to indicate biomarker positivity in SCD participants, which could be used as a safer and more cost‐effective variable for enriching clinical trials in AD.